# Imaging-Based Outcome Prediction of Acute Intracerebral Hemorrhage

**DOI:** 10.1007/s12975-021-00891-8

**Published:** 2021-02-06

**Authors:** Jawed Nawabi, Helge Kniep, Sarah Elsayed, Constanze Friedrich, Peter Sporns, Thilo Rusche, Maik Böhmer, Andrea Morotti, Frieder Schlunk, Lasse Dührsen, Gabriel Broocks, Gerhard Schön, Fanny Quandt, Götz Thomalla, Jens Fiehler, Uta Hanning

**Affiliations:** 1 Department of Diagnostic and Interventional Neuroradiology, University Medical Center Hamburg Eppendorf, Hamburg, Germany; 2 Department of Radiology, Charité - Universitätsmedizin Berlin, Campus Mitte, Campus Mitte, Humboldt-Universität zu Berlin, Freie Universität Berlin, Berlin Institute of Health (BIH), BIH Biomedical Innovation Academy, Berlin, Germany; 3 Department of Radiology, Charité School of Medicine and University Hospital Berlin, Berlin, Germany; 4 Department of Neuroradiology, Clinic for Radiology and Nuclear Medicine, University Hospital Basel, Basel, Switzerland; 5 Department of Radiology, University Hospital Muenster, Muenster, Germany; 6 Neurology Unit, ASST Valcamonica, Esine, BS Italy; 7 Department of Neurosurgery, University Medical Center Hamburg-Eppendorf, Hamburg, Germany; 8 Institute of Medical Biometry and Epidemiology, University Medical Center Hamburg-Eppendorf, Hamburg, Germany; 9 Department of Neurology, University Medical Center Hamburg-Eppendorf, Hamburg, Germany

**Keywords:** Intracerebral hemorrhage, Outcome prediction, Radiomics, Machine Learning

## Abstract

We hypothesized that imaging-only-based machine learning algorithms can analyze non-enhanced CT scans of patients with acute intracerebral hemorrhage (ICH). This retrospective multicenter cohort study analyzed 520 non-enhanced CT scans and clinical data of patients with acute spontaneous ICH. Clinical outcome at hospital discharge was dichotomized into good outcome and poor outcome using different modified Rankin Scale (mRS) cut-off values. Predictive performance of a random forest machine learning approach based on filter- and texture-derived high-end image features was evaluated for differentiation of functional outcome at mRS 2, 3, and 4. Prediction of survival (mRS ≤ 5) was compared to results of the ICH Score. All models were tuned, validated, and tested in a nested 5-fold cross-validation approach. Receiver-operating-characteristic area under the curve (ROC AUC) of the machine learning classifier using image features only was 0.80 (95% CI [0.77; 0.82]) for predicting mRS ≤ 2, 0.80 (95% CI [0.78; 0.81]) for mRS ≤ 3, and 0.79 (95% CI [0.77; 0.80]) for mRS ≤ 4. Trained on survival prediction (mRS ≤ 5), the classifier reached an AUC of 0.80 (95% CI [0.78; 0.82]) which was equivalent to results of the ICH Score. If combined, the integrated model showed a significantly higher AUC of 0.84 (95% CI [0.83; 0.86], *P* value <0.05). Accordingly, sensitivities were significantly higher at Youden Index maximum cut-offs (77% vs. 74% sensitivity at 76% specificity, *P* value <0.05). Machine learning–based evaluation of quantitative high-end image features provided the same discriminatory power in predicting functional outcome as multidimensional clinical scoring systems. The integration of conventional scores and image features had synergistic effects with a statistically significant increase in AUC.

**Supplementary Information:**

The online version contains supplementary material available at 10.1007/s12975-021-00891-8.

## Introduction

Intracerebral hemorrhage (ICH) is the most severe form of stroke with a 1-month morbidity and mortality approaching 50% and death or severe disability exceeding 75% [1–3]. In contrast to recent advances in interventional treatments of patients with ischemic stroke, beneficial effects of medical treatment and surgical intervention on the mortality and functional outcome of ICH patients were not observed in recent trials [4, 5]. Accurate stratification of ICH prognosis is highly desired regardless of the therapeutic options that are available and remains a clinical research priority [6]. Therefore, several prognostic tools have been proposed for the prediction of mortality and functional outcome in spontaneous ICH [7]. Though potentially useful for ascertaining prognosis and facilitating communication between clinicians, numerous methodological and reporting deficiencies are reported for a majority of these tools [7]. There is growing interest in augmented diagnostic and prognostic vision with machine learning (ML) in the medical field due to the wide range of applications of these algorithms and the increasing availability of computational power. ML is a type of artificial intelligence that learns patterns and rules from given information [8]. Recent studies applied ML to severity and outcome prediction models for neurological disorders such as ischemic stroke [8], aneurysmal subarachnoid hemorrhage [9], and traumatic brain injury [10]. However, ML approaches in the field of ICH were mainly focused on prompt diagnosis and automated volume quantification [11, 12] with lacking algorithms for the prediction of clinical outcome. As of late, Wang et al. have been among the first to develop an outcome prediction model based on ML by incorporating initial clinical presentations, laboratory data, and imaging findings [13]. Imaging findings were limited to ICH volume and location, presence of intraventricular hemorrhage, ventricle compression, and midline structure shift [13]. Further integration of quantitative imaging characteristics may hold additional prognostic value [9]. In the past, specific CT markers and histogram-based analyses of ICH heterogeneity have been linked to poor clinical outcome and reinforce this notion [14–16]. The goal of this study was twofold: First, we hypothesized that quantitative radiomic filter- and texture-derived high-end image features extracted from non-enhanced computed tomography (NECT) brain scans can be used to predict clinical outcome of ICH patients. To test and evaluate this hypothesis, we employed a radiomics-based ML approach on NECT brain scans of patients presenting with acute primary ICH [17]. Secondly, we hypothesized that the diagnostic power of the presented algorithm using high-end image features is equal to the ICH Score serving as the most widely utilized prognostic model for predicting mortality [18].

## Materials and Methods

### Study Population

We retrospectively analyzed the database of three university hospitals (University Medical Center Hamburg-Eppendorf, Charité University Medical Center Berlin, University Medical Center Münster) with a high-volume tertiary stroke center, for patients with ICH aged ≥18 years between January 2010 and April 2019. Inclusion criteria were defined as follows: Spontaneous ICH confirmed on NECT on admission. Patients were excluded if they had a secondary ICH from head trauma, hemorrhagic transformation of ischemic infarction, brain tumor, cerebral aneurysm, or vascular malformation. Baseline patient characteristics were retrieved from medical records, including Glasgow Coma Scale (GCS) at admission and modified Rankin Scale (mRS) at discharge. Additionally, we obtained vascular risk factors, blood pressure parameters, antiplatelet and oral anticoagulation (OAC) medication, and follow-up procedures, such as craniectomy or intraventricular drainage placement from patients’ clinical records and follow-up CT. A binary clinical outcome was defined based on modified Rankin Scale (mRS) on discharge with ≤3 as good outcome and mRS >3 as poor outcome [19]. According to the inclusion criteria, 520 patients were included, out of which 151 (29%) patients had a good outcome (mRS 0–3) and 369 (71%) patients had a poor outcome (mRS 4–6). Details are listed for further consideration in Table 1. This multicenter retrospective study was approved by the ethics committee (Ethik-Kommission der Ärztekammer Hamburg, Ethik-Komission der Charité Berlin) and written informed consent was waived by the institutional review boards. All study protocols and procedures were conducted in accordance with the Declaration of Helsinki. The deidentified data and analytic code are available from the corresponding author upon reasonable request.

**Table 1:** Baseline demographic, clinical, and radiological characteristics of study cohort

Baseline characteristics	All (*n* = 520)	mRS 0–3 (*n* = 151)	mRS 4–6 (*n* = 369)	*P* value
Clinical parameters				
Age [years], median (IQR)	73 (59; 79)	70 (57; 78)	73 (60; 80)	0.85
Female, *n* (%)	234 (45.0)	67 (44.4)	167 (45.3)	0.85
Hypertension, *n* (%)	359 (69.2)	99 (65.6)	260 (70.7)	0.25
Diabetes mellitus, *n* (%)	73 (14.0)	23 (15.2)	50 (13.7)	0.62
Antiplatelet medication, *n* (%)	104 (20)	33 (21.9)	71 (19.2)	0.50
Anticoagulant medication, *n* (%)	113 (21.7)	34 (22.5)	79 (21.4)	0.78
Systolic blood pressure [mm Hg], median (IQR)	162 (138; 193.75)	162 (145; 185)	160 (135; 197.5)	0.75
Time from symptom onset to CT [days], median (IQR)	0.19 (0.76; 0.52)	0.21 (0.09; 0.59)	0.19 (0.07; 0.52)	0.92
Time from CT to discharge [days], median (IQR)	14 (7; 22)	14 (6.5; 18.5)	15.5 (6.75; 23.5)	0.13
Clinical scores				
GCS Score, median (IQR)	11 (5; 14)	14 (12; 15)	9 (3; 13)	<0.001
ICH Score, median (IQR)	2 (1; 3)	1 (0; 2)	3 (2; 4)	<0.001
CT parameters				
Bleeding location, *n* (%)				
- Lobar - Basal ganglia - Thalamus - Brainstem and pons - Cerebellar	238(45.8) 198 (38.1) 18 (3.5) 23 (4.4) 43 (8.3)	76 (50.3) 54 (35.8) 3 (2.0) 5 (3.3) 13 (8.6)	162 (43.9) 144 (39.0) 15 (4.1) 18 (4.9) 30 (8.1)	0.18 0.49 0.24 0.43 0.86
Intraventricular hemorrhage, *n* (%)	267 (51.3)	50 (33.1)	217 (59)	<0.001
ICH volume [mL], median (IQR)	25.1 (9.7; 60.3)	11.5 (3.6; 24.7)	35.5 (14.9; 73.2)	<0.001
Surgical procedures				
Craniectomy, median (IQR)	117 (22.5)	16 (10.6)	101 (27.4)	<0.001

Comparison of baseline demographic, clinical, and radiological characteristics between ICH patients with good clinical outcome (modified Rankin Scale (mRS) 0–3) versus poor clinical outcome (mRS 4–6). ICH Score, Intracerebral Hemorrhage Score; *GCS*, Glasgow Coma Scale; *IQR*, interquartile range

### Image Acquisitions

The NECT scans were performed using standard clinical parameters with axial < 5 mm section thickness. All datasets were inspected for quality and excluded in case of severe motion artifacts. In detail, the images were acquired on the following scanners: 256 slice scanner (Philips iCT 256) with 120 kV, 280–320 mA, < 5.0 mm slice reconstruction; 80 slice scanner (Toshiba Aquilion Prime) with 120 kV, 280 mA, < 5.0 mm slice reconstruction and < 0.5 mm in-plane resolution; and 2 × 128 slice scanner (SOMATOM Definition Flash) with 120 kV, 280 mA, < 5.0 mm slice reconstruction and < 0.5 mm in-plane resolution.

### Post-procedure Evaluations

NECT scans were obtained and stored for further evaluation. Two experienced neuroradiologists (JN and SE) assessed and documented the following imaging features on NECT scans: [1] intraventricular hemorrhage; [2] ICH location; [3] craniectomy in the follow-up NECT scans. ICH locations were classified as basal ganglia, thalamus, lobe, brain stem, pons, and cerebellum. In the following ICH, volumes were segmented semi-automatically on the basis of the original NECT images [20]. Regions of interest (ROIs) were delineated using Analyze 11.0 Software (Biomedical Imaging Resource, Mayo Clinic, Rochester, MN). Consensus ROIs were derived based on overlapping segmentations of both readers. Both readers were blinded to all clinical information and bleeding location. Discrepancies were settled by joint discussion of the 2 readers and a third reader (UH). JN and SE: 3 years clinical experience in diagnostic neuroradiology in an academic full-service hospital; UH: 8 years clinical experience in diagnostic neuroradiology; JN, SE, and UH: research with focus on clinical applications of image processing and predictive modelling.

### ICH Score

ICH Scores were obtained for every patient included according to the definition of Hemphill et al. based on five independent and multidimensional predictors (ICH volume, infratentorial location, GCS, age, and intraventricular extension) [18]. ICH volumes were obtained from ICH delineations. Oral anticoagulants (OAC) were not included as their addition does not increase the prognostic performance of the ICH Score [21]. As the ICH Score is a prognostic model for 30-day mortality in ICH patients (equivalent to mRS 6), a binary mortality outcome was defined based on mRS at discharge with mRS ≤ 5 (survival) and mRS = 6 (death).

### Imaging-Based Outcome Prediction

Radiomic features were defined according to the PyRadiomics Python package v2.1.0. Features were extracted from consensus ROIs and resampled to 0.5 mm × 0.5 mm × 2 mm resolution using sitk BSpline interpolators. Resampling was performed to ensure comparability of texture analysis. Extracted features comprised 252 first-order features (thereof 18 based on unfiltered images, 144 based on wavelet decompositions, 90 based on log-sigma laplacian of Gaussian filters), 902 texture features (thereof 68 based on unfiltered images, 544 based on wavelet decompositions, 290 based on log-sigma laplacian of Gaussian filters), and 14 shape features. In total, 1218 quantitative image features were extracted from the ICH ROIs. To adjust for effects of therapeutic interventions that cannot be detected on admission NECTs, we included decompressive craniectomy as sole clinical parameter into the machine learning models.

ML-based classification was performed using random forest algorithms (Python scikit-learn environment v0.20.3 [22]). Random forest is a ML technique that utilizes multiple decision trees trained on random sub-selections of samples in order to improve stability and reduce overfitting of the algorithm [23]. Decision trees learn decision rules according to predictor values of the training data samples. With increasing depth of nodes, decision trees can represent more complex decision rules, resulting in a better fitting of the model [23, 24]. Hyperparameter tuning (total number of features, number of trees, maximum depth of the tree, minimum number of samples to split an internal node, number of features considered for splitting (m_try_), minimum number of samples at leaf node, bootstrapping yes/no) was performed in a nested 5-fold cross-validation approach for each training set using grid search algorithms. Parameters at initiation were set to scikit-learn default values.

Selection of features with highest predictive value was conducted separately for each training dataset of the 5-fold cross-validation outer loop sample split according to Gini impurity measures [25]. Classifier models were trained and tested on each set’s unique training and testing samples (outer loop) utilizing optimized hyperparameters and feature importance of the respective training data (inner loop).

### Integration of ICH Score and Imaging-Based Outcome Prediction

It was shown that combinations of classification models trained on heterogeneous predictors tend to have higher synergistic effects if knowledge flows are merged at a very late stage of the data evaluation process. Therefore, probabilities for survival of the ICH Score and of the imaging-based classifier were extracted. The arithmetic average of both probabilities was then used for outcome prediction.

### Statistics

Model validation and testing of all classifiers was conducted in a nested 5-fold cross-validation with independent training and validation sets in a model-external approach [26]. Accordingly, model selection and hyperparameter tuning was performed with grid search algorithms on each training data set using a second cross-validation layer. Model stability was examined through comparative analysis of 10 randomly permuted cross-validation sets.

Receiver-operating characteristic (ROC) curves were generated from prediction results of all cross-validation sets. Confidence intervals (CI) for sensitivities and specificities were bootstrapped (2000 replicates, pROC v1.15 [27] R-package). Bonferroni adjustments were applied to control for alpha error inflation.

Furthermore, the classifiers were analyzed using ROC areas under the curve (AUC), sensitivity, specificity, accuracy, Youden Index, positive predictive value, negative predictive value (ThresholdROC v2.8 R-package), and Matthews correlation coefficient (MCC) [28] metrics (psychometric v.2.2. R-package). MCC evaluates all fields of the confusion matrix and is considered a favorable measure for unbiased comparisons of binary classifiers [29]. With *TP*: true positives, *TN*: true negatives, *FP*: false positives, and *FN*: false negatives, MCC is defined as:

$$ MCC=\frac{TP\ x\  TN- FP\ x\  FN\ }{\sqrt{\left( TP+ FP\right)\left( TP+ FN\right)\left( TN+ FP\right)\left( TN+ FN\right)}} $$

**Fig. 1:**
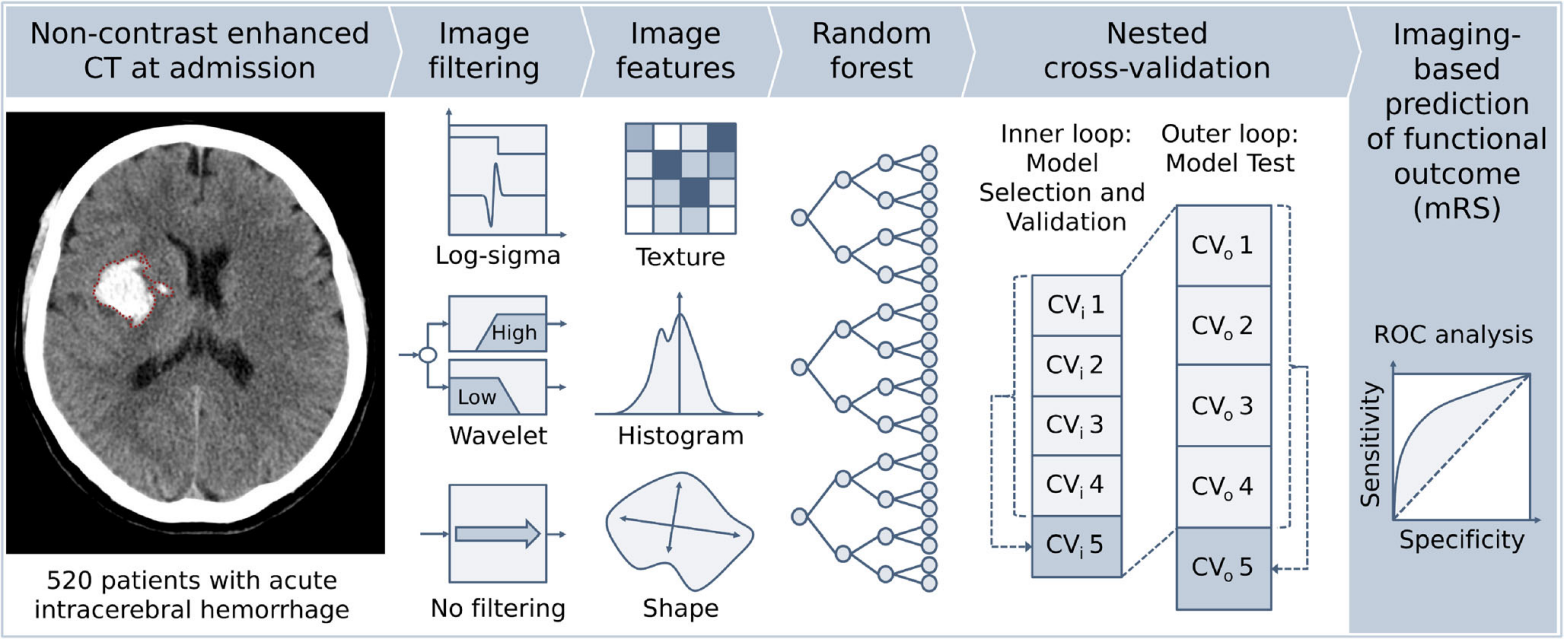
Conceptual overview of the proposed machine learning approach for intracerebral hemorrhage outcome prediction showing the major processing steps: CT based image acquisition and segmentation, feature extraction (*n* = 1218), and statistical learning (random forest algorithm). NECT, non-contrast-enhanced computed tomography; ICH, intracerebral hemorrhage; CT, computed tomography; mRS, modified Rankin Scale; CV, cross-validation set with i: inner loop and o: outer loop

A flow chart of the proposed ML-based prediction of the clinical outcome is depicted in Fig. 1.

## Results

Our analysis included NECT images of 520 patients with acute ICH. One hundred fifty-one patients (29%) had a mRS of 0–3 and 369 (71%) had a mRS of 4–6. There were no statistically significant differences in clinical parameters age (*P* value = 0.85), sex (*P* value = 0.85), hypertension (*P* value = 0.25), diabetes mellitus (*P* value = 0.62), antiplatelet or anticoagulant medication (*P* value = 0.5 and *P* value = 0.78, respectively), and systolic blood pressure at admission (*P* value = 0.75). Both time from symptom onset to admission CT and time from CT to hospital discharge were not statistically different (*P* value 0.92 and *P* value = 0.13, respectively). However, patients with mRS 4-6 had a significantly lower GCS (GCS 9 versus GCS 14; *P* value <0.001), higher percentage of intraventricular hemorrhage (59% versus 33.1%; *P* value <0.001), higher ICH volumes (35.2 cm^3^ versus 8.4 cm^3^; *P* value <0.001), and a higher rate of supra-tentorial craniectomies (27.4% versus 10.6%; *P* value <0.02). There were no significant differences in ICH locations. ICH Score was significantly higher in patients with mRS 4-6 (median 3 versus 1; *P* < 0.001).

### Imaging-Based Outcome Prediction

**Fig. 2:**
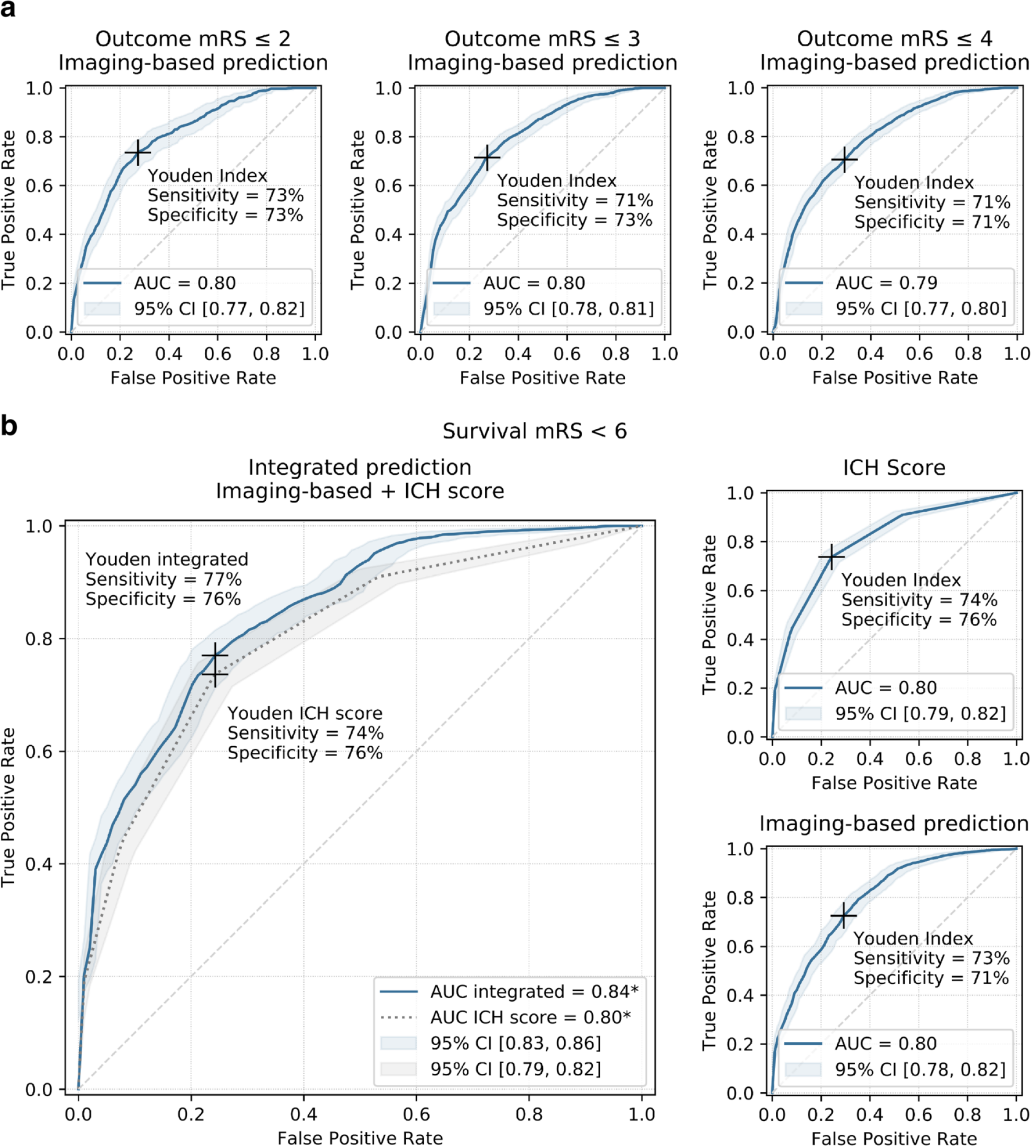
Receiver-operating characteristics (ROC) curves for (**a**) functional outcome prediction of the proposed machine learning classifier based on quantitative image features and (**b**) prediction of survival using the ICH Score, the proposed machine learning classifier based on quantitative image features, and a classifier integrating ICH Score metrics and quantitative image features. AUC, area under the curve; CI, confidence interval; mRS, modified Rankin Scale

**Fig. 3:**
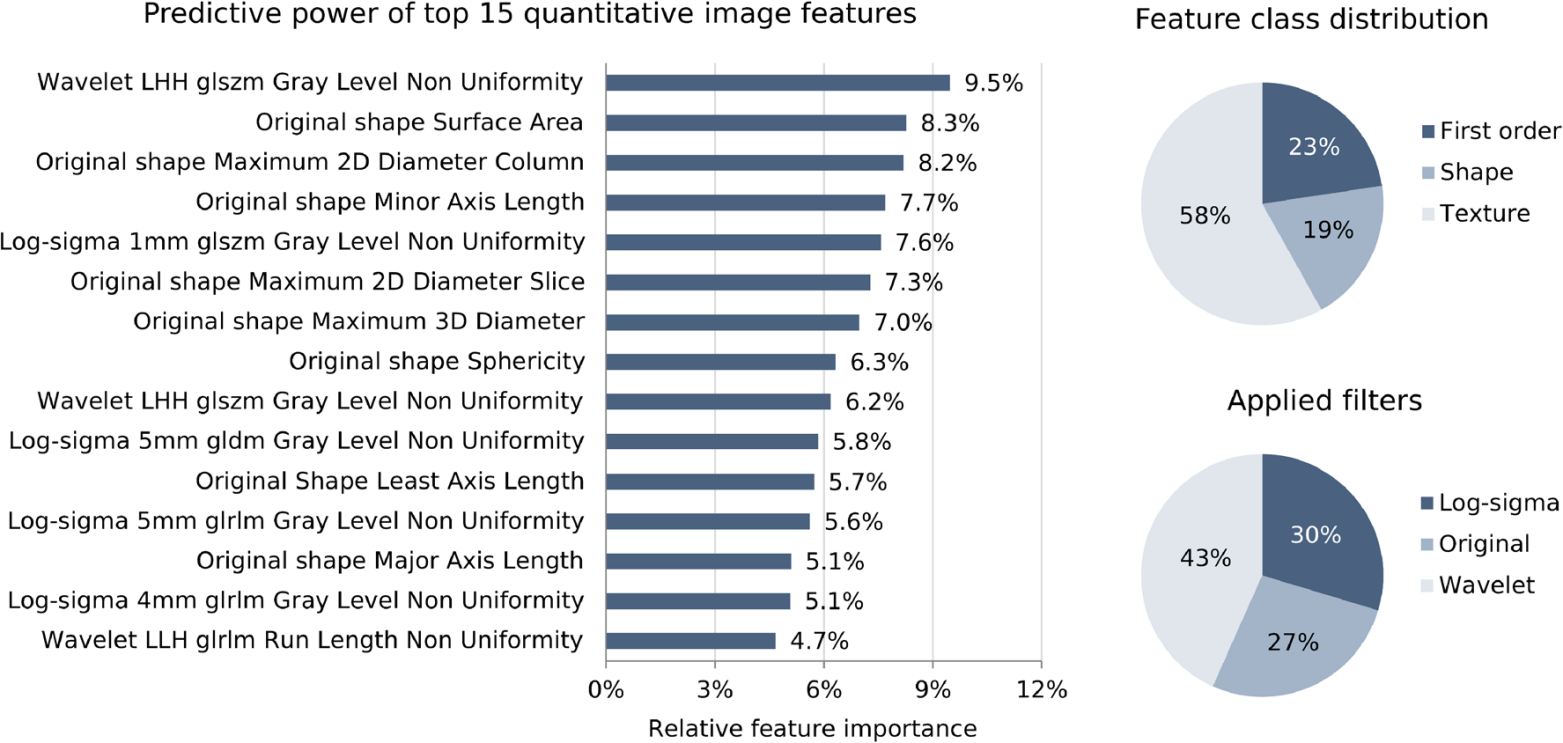
Predictive value of quantitative image features. Bar charts show mean Gini impurity feature importance of all cross-validation training sets of the top- 15 high-end image features. Pie charts show distribution of feature classes and applied filters in utilized top-100 predictors. First-order metrics: Basic statistical metrics of the voxel grey level distribution; glcm: gray level co-occurrence matrix; gldm: gray level dependence matrix; glrlm: gray level run length matrix; glszm: gray level size zone; H: high-pass wavelet decomposition; L: low-pass wavelet decomposition

Machine learning–based ROC AUCs of the validation sets for predicting functional clinical outcome were 0.80 (95% CI [0.77; 0.82]) for mRS ≤ 2, 0.80 (95% CI [0.78; 0.81]) for mRS ≤ 3, and 0.79 (95% CI [0.77; 0.80]) for mRS ≤ 4. Trained on survival prediction (mRS ≤ 5), the classifier reached ROC AUCs of 0.80 (95% CI [0.78; 0.82]) which was equivalent to results of the ICH Score with ROC AUC of 0.80 (95% CI [0.79; 0.82]) (Fig. 2, Table 2). Exclusion of the parameter craniectomy yes/no had no effect on classification performance. Model selection and hyperparameter tuning within the nested cross-validation process resulted in the following median settings for mRS ≤ 2, ≤ 3, ≤ 4, and ≤ 5, respectively (medians over cross-validation sets): Number of features considered: 25, 100, 200, 100; number of trees: 750, 1000, 500, 1000; maximum depth of trees: 10 for all cut-off values; number of features considered for splitting (m_try_), minimum number of samples to split an internal node, and minimum number of samples at leaf node: 1 for all cut-off values. Feature importance analyses of the mean top 100 predictors of all training data sets suggests that features with highest predictive power are mainly derived from wavelet (43%) and log-sigma (30%) filtered images. Unfiltered original images contributed 27% to total predictive power. Within feature classes, texture metrics dominated predictions (58%) (Fig. 3). Predictive power of the 15 most important features demonstrates dominance of texture and shape features compared to first-order metrics (basic statistical measures of the grey level distribution). To also assess the predictive value of the ICH volume only, an additional ROC analysis was performed (supplementary Figure 1). ROC AUC for ICH volume as sole predictor was 0.72 with a Youden Index of 0.30 at 60% specificity and 70% sensitivity.

**Table 2:** Classification performance of imaging-based outcome prediction

Prediction mRS	Patients (*n*; %)	AUC [95% CI]	MCC max [95% CI]	Sensitivity [95% CI]	Specificity [95% CI]	Accuracy [95% CI]	PPV [95% CI]	NPV [95% CI]
A) Imaging-based ML prediction of functional outcome (mRS)								
mRS ≤ 2	75/520(14%)	0.8[0.77; 0.82]	0.36[0.33; 0.39]	73%[68%; 77%]	73%[72%; 75%]	73%[72%; 75%]	31%[28%; 35%]	94%[93%; 95%]
mRS ≤ 3	151/520(29%)	0.8[0.78; 0.81]	0.42[0.39; 0.45]	71%[67%; 74%]	73%[71%; 75%]	72%[71%; 74%]	52%[49%; 55%]	86%[84%; 88%]
mRS ≤ 4	259/520(50%)	0.79[0.77; 0.8]	0.47[0.44; 0.5]	71%[68%; 73%]	71%[68%; 73%]	71%[69%; 72%]	70%[68%; 73%]	71%[68%; 73%]
B) Imaging-based ML, ICH Score, and integrated model-based prediction of survival (mRS ≤ 5)								
Imaging-based ML classifier	375/520(72%)	0.8[0.78; 0.82]	0.46[0.43; 0.49]	73%[70%; 75%]	71%[67%; 74%]	72%[70%; 74%]	87%[85%; 88%]	50%[47%; 53%]
ICH Score	375/520(72%)	0.80*[0.78; 0.82]	0.46[0.43; 0.49]	74%*[72%; 75%]	76%[73%; 79%]	74%[72%; 76%]	89%[87%; 90%]	53%[50%; 56%]
Image features + ICH Score	375/520(72%)	0.84*[0.86; 0.83]	0.49[0.46; 0.52]	77%*[75%; 79%]	76%[72%; 79%]	77%[75%; 78%]	89%[87%; 91%]	56%[53%; 59%]

*P value <0.05

Prediction of outcome in patients with acute ICH: Number of patients with respective outcome (positive class) and performance metrics of A) imaging-based random forest machine learning classification at different mRS levels and B) ICH Score–based prediction compared to an integrated model using knowledge derived from both the machine learning algorithm and the ICH Score. Metrics are shown at Youden Index maximum cut-off points. Bonferroni corrections have been applied to account for alpha spending error. Results are based on nested 5-fold cross-validation of 520 patients from three different centers. *AUC*, receiver-operating-characteristic area under the curve; *PPV*, positive predictive value; *NPV*, negative predictive value; *MCC*, Matthews correlation coefficient; *CI*, confidence interval; *mRS*, modified Rankin Scale

### Integration of ICH Score and Imaging-Based Outcome Prediction

ICH Score metrics reached a ROC AUC of 0.80 (95% CI [0.79; 0.82]), which was equivalent to the purely imaging-based classifier with ROC AUC of 0.80 (95% CI [0.78; 0.82]). If combined, the integrated model showed a significantly higher ROC AUC of 0.84 (95% CI [0.83; 0.86], *P* value <0.05). Sensitivities of the integrated model were significantly higher at Youden Index maximum cut-offs with 77% vs. 74% sensitivity at 76% specificity, *P* value <0.05 (Fig. 2, Table 2).

## Discussion

In this study, we developed an imaging-based ML model for predicting the functional outcome of ICH patients. The proposed approach employing quantitative image features derived from NECT scans provided high discriminatory accuracy between good and poor functional outcome of ICH patients at different mRS cut-off values. This study is based on a large multicenter and heterogeneous imaging dataset of 520 patients that was acquired in clinical routine over almost a decade. The proposed classification is solely based on high-end image features without a priori information about the location of the hemorrhage and without controlling for factors such as patient conditions, image acquisition parameters, or scanner type. Observed classification performance and model stability across all nested cross-validation runs suggest sufficient generalizability of our results.

It is a well-known paradigm that the ICH volume profoundly impacts functional clinical outcome. Initially derived by Broderick et al. to predict 30-day mortality after ICH, the ICH volume has been later validated and included in the ICH Score [3, 18]. In line with these findings, we have shown that ML-based outcome assessment using ICH volume as sole predictor already achieves ROC AUCs of >0.70 (supplementary Figure 1). Similarly, surrogate parameters of ICH volume such as maximum 2D diameter or minor axis length had comparatively high predictive importances in the imaging-based ML model. However, total contribution to predictive power of shape-based metrics in the comprehensive model was only 19% at ROC AUCs of 0.80. It thus stands to reason that the ICH formation on NECT holds additional and relevant information which is not assessable by human eyes but can be evaluated by imaging-based ML algorithms. As so, analyses of the 100 most powerful features demonstrate the importance of second-order features (e.g., texture metrics) in comparison to first-order features. In contrast to first-order measures, second-order metrics also capture information regarding the spatial distribution of gray levels and are often difficult to evaluate by the human visual system. The predictive value of second-order features is particularly apparent in the high predictive power of the gray level non-uniformity (Fig. 3). This specific finding could be related to the heterogenous appearance of hematomas that are still actively bleeding with evidence of spot sign or in those of patients with anticoagulation that are at risk for further expansion. It is equally conceivable that the gray level non-uniformity may differentiate areas of hyperacute ICH as the blend sign—with blending of a hypoattenuating area and a hyperattenuating region relative to the surrounding brain parenchyma—suggesting hematoma expansion and in reversal poor clinical outcome.

Hence, the proposed approach can be used as supportive tool to augment conventional image analysis and to improve prognostic decision for both radiologists and clinicians. As aspects of precision medicine are an emerging concept [30], combining the ICH Score with high-end imaging features may be useful in this respect. In line with this, the ICH Score seems to be limited in extension to critical care patients. In a prospective multicenter cohort study with patients presenting with spontaneous ICH and admitted to the intensive care unit (ICU), the ICH Score had only acceptable discriminatory power [31]. Although at this stage speculative and part of future studies, the proposed ML classifier may provide promising complementary results. In anticoagulation-associated ICH, the ICH Score may not be as reliable [21, 32, 33] and clinical outcomes in these patients likewise substantially often worse in comparison to patients without oral anticoagulation (OAC) [34, 35]. Assuming that OAC therapy alters morphology and intensity of ICH, it is most likely that radiomic features are affected by OAC therapy. As we trained the ML model on acute CT images of both, patients receiving OAC and patients without OAC, the information on OAC therapy is incorporated in the model through these differences in ICH imaging characteristics.

Since our quantitative imaging feature analysis performs equally in comparison to multidimensional scoring systems (e.g., ICH Score), the application of the proposed ML approach may be of value for randomized clinical trials. Challenges and opportunities to optimize clinical research and randomized trials in ICH are ongoing [36]. The ML approach could simplify trial procedures by performing an imaging-based prediction of functional outcome or early mortality. Simultaneously the multicenter approach of this study takes local variations in practice into account which are necessary to reflect upon a successful trial planning. Furthermore, this approach may also be of value for telemedicine and remote prediction of ICH outcome in regions lacking neuroradiological specialists. Taken together, the proposed method integrates the merits from quantitative radiomic features and ML algorithms and relates the employed predictors to well-known imaging characteristics.

Despite the promising results, several limitations deserve comment. Our study had general limitations typically associated with quantitative radiomics-based image analysis and classification [17, 37–39]. These limitations include differences in image acquisition settings (e.g., size of the field of view, gantry tilt) and under- or overfitting of machine learning algorithms. Bias of these factors was minimized through (a) employment of NECT scans that offer standardized HU metrics and (b) the application of random forest algorithms that are comparably stable with regard to overfitting. The risk of overfitting was further reduced by evaluating multiple different models in a nested cross-validation approach. Furthermore, we observed study-specific limitations: First, we included a limited number of patients in a retrospective analysis. An expansion of sample size in a prospective study design would certainly contribute to further improving generalizability of our results. However, observed model stability suggests sufficient robustness for evaluating feasibility and limitations of the proposed algorithm. The utilized dataset includes imaging data from 520 patients acquired over a relatively long period of almost a decade in three different centers. In such heterogeneous datasets, results of nested cross-validation approaches serve as a valid indicator for confirming feasibility and performance of the proposed classifier in the underlying clinical setting. Due to standardized and calibrated quantitative imaging parameters and signal intensity processing of CT scanners, we assume neglectable bias on classifier performance in a generalized setting. Second, the manual definition of ROIs still implies a certain degree of observer dependence within the ML process. To minimize its influence, we employed consensus segmentations from two independent readers and applied a semi-automated delineation method that was shown to have a favorable inter- and intra-observer reliability and a high level of congruence with a fully automated delineation [20, 40]. Furthermore, it was found that radiomic features are relatively stable with regard to variations in segmentations [41, 42]. The lack of data on withdrawal and limitation of care are a further limitation [43]. Final limitation was the missing correlation with long-term data (e.g., mRS at 90 days and mortality) as it might offer additional information but was not available for this study [44].

## Conclusion

Quantitative imaging features of acute NECT evaluated by ML algorithms provide a high discriminatory power in predicting functional outcome in patients with spontaneous ICH. Additional integration of the ICH Score increases predictive power of the ML classifier, hence providing promising complementary results. The findings support the potential of ML algorithms to augment conventional image analysis, improve prognostic decision, and simplify trial procedures. In the very near future, such ML techniques may play a pivotal role in determining optimized therapeutic regimes and predicting the prognosis for patients with ICH in an individualized manner.

## Supplementary Information

ESM 1 (DOCX 25 kb)
