# Supplementary material for: Imaging-Based Outcome Prediction of Acute Intracerebral Hemorrhage
Source: Transl Stroke Res. 2021 Feb 6;12(6):958–67. doi: 10.1007/s12975-021-00891-8 (PMC8557152; doi:10.1007/s12975-021-00891-8)

**Supplementary Material**

**Figure 1:** Receiver Operating Characteristics (ROC) curves of functional outcome prediction of the proposed machine learning classifier based on continuous ICH volume only. ICH: Intracerebral hemorrhage. Area under the curve; CI: confidence interval; mRS: Modified Rankin Scale.


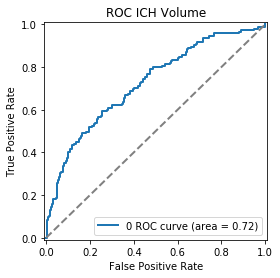

Supplement: Supplementary file 1 — (DOCX 25 kb) [file 12975_2021_891_MOESM1_ESM.docx]
